# Predictors of Post Pericardiotomy Low Cardiac Output Syndrome in Patients With Pericardial Effusion

**DOI:** 10.15171/jcvtr.2015.04

**Published:** 2015-03-29

**Authors:** Feridoun Sabzi, Reza Faraji

**Affiliations:** ^1^ Department of Cardiovascular Surgery, Imam Ali Heart Center, Kermanshah University of Medical Sciences, Kermanshah, Iran; ^2^ Yazd Cardiovascular Research Center, Shahid Sadoughi University of Medical Sciences, Yazd, Iran

**Keywords:** Disease, Pericardium, Low Cardiac Output Syndrome

## Abstract

***Introduction:*** Pathological involvement of pericardium by any disease that resulting in effusion may require decompression and pericardiectomy. The current article describes rare patients with effusion who after pericadiectomy and transient hemodynamic improvement rapidly developed progressive heart failure and subsequent multi organ failure.

***Methods:*** During periods of five years, 423 patients in our hospital underwent pericardiotomy for decompression of effusion. The clinical characteristics of those patient with postoperative low cardiac output (B group) (14 cases) recorded and compared with other patients without this postoperative complication (A group) by test and X2. Significant variables in invariables (P≤0.1) entered in logistic regression analysis and odd ratio of these significant variables obtained.

***Results:*** Idiopathic pericardial effusion, malignancy, renal failure, connective tissue disease, viral pericarditis was found in 125 patients (27%), 105 patients (25.4%), 65 patients (15.6%), 50 (17.1%) and 10 (2.4%) of patients subsequently. The factors that predict post-operative death in logistic regression analysis were malignancy, radiotherapy, constrictive pericarditis inotropic drug using IABP using, pre-operative EF and pericardial calcification.

***Conclusion:*** Certain preoperative variables such as malignancy, radiotherapy, low EF, calcified pericardium and connective tissue disease are associated with POLCOS and post-operative risk of death. This paradoxical response to pericardial decompression may be more frequent than currently appreciated. Its cause may relate to the sudden removal of the chronic external ventricular support from the effusion or thicken pericardium resulting in ventricular dilatation and failure or intra operative myocardial injury due to pericardiectomy of calcified pericardium, radiation and cardiomyopathy.

## Introduction

Pericardiectomy or pericardiotomy is easy but difficult surgery. Every experienced cardiac surgeon confronted with rare, unexpected and fulminant heart failure after pericardiectomy or pericardiotomy for effusion or constrictive pericarditis. Low cardiac output for first time described by Vandyke et al.^1^ following pericardioyomy for cardiac tamponade. Vandyke^1^ revealed that cardiac dysfunction following tamponade decompression may be related to acute hemodynamic changes related to change of intraventricular volume in the setting of dilated ventricles, and changing systemic vascular resistance. The Frank-Starling mechanism is improved initially by myocardial response to the release of constrictive pericarditis and improvement of right ventricular ejection fraction accompanying pericardiectomy but further increasing of intraventricular volume causes increasing systolic wall stress, a reduction in stroke volume and low cardiac output. Spodick^2^ and Glasseret al^3^ have revealed that this response may be related to the magnitude and the velocity at which the load develops. Chamoun et al^4^ have hypothesized that an imbalance between the sympathetic and parasympathetic output of the autonomic system, with an apparent attenuation of sympathetic outflow (following relief of tamponade) unmasking occult left ventricule (LV) dysfunction and caused post low cardiac output syndrome (POLCOS) however Wolfe and Edelman^5^ rule out this hypotesis by this issue that complete recovery of LV function occurs in most patient after pericardiectomy. Hamaya et al^6^ attributed this syndrome to myocardial stunning as results of changing intramyocardial blood distribution during tamponade surgery. Wechsler et al^7^ proposed that subendocardial hemorrhage during tamponade drainage caused myocardial stunning and necrosis. Ligero et al^8^ does not attributed post pericardiotomy transient biventricular dysfunction to main coronary artery disease. In Wolfe and Edelman study^5^ two important and predicting factors are abrupt increase in myocardial wall stress, and time of the chronicity of tamponade. Wechsler et al^7^ proposed that homogeneity of two dimensional strain measurements shows that myocardial stunning may play a contributory role, considering the almost complete and uniform recovery of function in most patients. In the studies of Gregory et al^9^ and Dosius and Angouras^10^, low cardiac output as a post operating feature of decompressed tamponade was rare, ranging from 4.1% to 7.5% of hospitalized patients. Moores et al^11^ reported that the in hospital mortality after subxiphoid pericardiectomyis stated to be 20% that are reported to be unrelated to the surgical procedure. The 24% post-operative mortality in Gregory et al^9^ study in patients undergoing pericardiectomyalso attributedtothe low cardiac output.Another proposal that has been showed by Palatianos et al^12^ is that occultsystolic dysfunction may already be present during tamponadebut it may be masked by reduced chamber sizes and the tachycardia.The dysfunction is then brought out by pericardial decompression.Alternatively, high levels of sympathetic tone and endogenouscatecholamines during tamponade may mask preexisting myocardialdysfunction, which is then accentuated after pericardial decompression.However, chronic preexisting impaired cardiac function is unlikelyas patients who survive this syndrome subsequently regain normalfunction. Braverman and Sundaresan^13^ proposed that however it is reasonable that decompression or removal of effusion caused hemodynamic improvement immediately in postoperatively period but in rare patients, paradoxical response with sever ventricular dysfunction occurs despites improvement of cardiac filling and stroke volume. McCaughan et al^14^ revealed that risk of postoperative low cardiac output after constrictive pericarditis is seen in up to 28% of patients opposed to 4.1 to 7.1% in non constrictive pericardial effusion. Douglas^15^ proposed that post operative low cardiac output in this setting related to myocardial atrophy. Robinson et al^16^ related this complication to rapidly over dilation of heart as results of releasing of chronic compression of heart by effusion and tight pericardium however gradual decompression of effusion may have some merit and prevent this lethal complication. Similarly, little data about decompensated heart failure in post operative surgery for pericardiotomy or pericardiectomy are available, even in referee center registries. It has been established that constrictive pericarditis patients have more diastolic and systolic dysfunction than non constrictive patients, including that caused by myocardial ischemia, stunning, atrophy or over dilatation. However, clinical presentation and outcomes of these patients with heart failure and depressed ventricular ejection fraction have not been studied in post pericardiectomy period. Therefore, the purpose of this study was to identify predictors of low cardiac output and mortality in decompensated severe heart failure in post pericardiectomy period.

## Materials and methods

We selected a cohort of patients hospitalized for decompersation of tamponade or constrictive pericarditis between 2007 and 2012, referred to a regional cardiac surgery hospital. Inclusion criteria were urgent surgery for tamponade or emergent or elective surgery for constrictive pericarditis. Exclusion criteria were preoperative successful pericardiocentesis, or redo operation. 423 patients entered in the study. The patients with respect to POLCOS divided in two groups and theirs preoperative, operative and post operative variables compared with t test and x2 variables. Continuous variables are expressed as mean and standard deviation, and were compared by using the Student t test. Categorical variables are expressed by number and proportion and compared by using the chi‐square test. We considered P < 0.05 (2‐tailed) as significant. Predictors of low cardiac output were defined by logistic regression and they are expressed by relative risk and 95% confidence interval. The surgical technique utilized was similar to that outlined by Fontenelle et al^17^ in 1970. No local anesthetics were used before or during the operation. In all patients, the procedure was performed under general endotracheal anesthesia after the patient was draped and the surgical team was prepared to commence the operation. For induction to anesthesia, fentanyl, and pancuronium or cis-atracurium was administered. Maintenance of anesthesia was obtained by isoflurane or fentanyle and mixture of oxygen/air supplemented by fentanyl as needed. The xiphoid process was excised. A piece of pericardium of approximately 2 cm by 5 cm was removed from the lower portion of the anterior surface of the pericardium. The usual duration of the operation was 35 to 50 min. Low cardiac output was defined based on Nohriaet al^18^ definition as congestion signs or without congestion signs profiles in clinical hemodynamic assessment compromised perfusion was assessed by the presence of a narrow pulse pressure, symptomatic hypotension, cool extremities, impaired mental function, or a combination of these.

### LCOS management routine

After definition of the clinical‐hemodynamic profile for each patient, those with low output signs were primarily treated with intravenous inotropic support: dobutamine, milrinone, and then by IABP, diuretic and intravenous furosemide.

## Results

Malignancy, uremia, and possible viral infections were the most common causes of massive pericardial effusions and cardiac tamponade. Idiopathic pericardial effusion was found in 125 patients (29.5%). In patients with tamponade, 105 patients (24.8%) had pericardial effusion due to malignancy. In the other 10 patients (2.36%), pericardial tamponade was the 1st manifestation of the disease, and causal investigations revealed the hypothyroidism. Uremic pericardial effusion was found in 65 patients (15.3%); 90.7% of these patients had been in routine dialysis. Viral pericarditis was the probable cause of pericardial effusions in 2.36% of all cases (10 patients). Seventy percent of the patients were subjectedto the operation because of clinically evident or impendingcardiac tamponade and the remaining 30% because of pericardialeffusion that was chronic, recurrent, or resistant to medicaltreatment. Twenty five (5.9%) patients were found to have malignanteffusions. The operative mortality, defined as death within30 days of the operation or during the same hospitalization,was 7% (30 patients), two of thirty deaths occurring inpatients with cancer and one in a patient with right atrial angiosarcoma and another in a patient with lymphoma. 410 of the 423 patients who were dischargedfrom the hospital were followed up regularly with clinical andechocardiographic examinations from 1 to 12 months. Five of these patients (1.18%) had a recurrence of the effusionnecessitating further surgical intervention. The diagnosis of viral pericarditis was assigned when the patient had a history of upper respiratory tract infection within 15 days before the onset of tamponade and when other causal factors could be excluded. In 10 patients (2.36%) pericardial effusions were due to systemic lupus erythematus. Diagnoses were confirmed by anti-DNA positivity. The other 40 patients (9.4%) had pericardial effusions due to others connective tissue disorders (Tables 1-3). Tuberculosis was the cause of pericardial effusion in 15 patients. Ziehl-Neelsen staining or culture of the pericardial fluid and tissue sample showed acid-resistant bacteria. Dressler syndrome was detected in 9 patients (1.8%). In the three of these patients, pericardial effusion occurred 1 month after myocardial infarction, and in the other six patients it occurred 2 months after myocardial infarction. In 12 patients (2.8%) the pericardial effusion was due to purulent pericarditis and *Staphylococcus aureus* and pneumococcus was grown in the culture. We could not identify the specific cause of pericardial effusion in 125 of the patients (29.5%). Five patients in B group died with postoperative low cardiac output. The one patient with pericardial tamponade that developed after lymphoma treatment and another with right atrial angiosarcoma died of low cardiac output on the operation day after successful surgical pericardiocentesis. One patient with tuberculosis constrictive pericarditis and one patient with purulent pericardial effusion died with postoperative heart failure. The fifth patient with POLCOS had systemic lupus erytmatus. Twenty five patients in A Group died within 7 days after surgical pericardiotomy because of respiratory failure, renal failure, gastrointestinal bleeding, stroke and pulmonary emboli. Postoperatively, most of the patients with POLCOS had hypotension, was inotrope dependent, and required an intra-aortic balloon pump. A TEE echocardiogram showed severe global hypo kinesis of the left ventricle or both ventricular with no regional wall motion abnormalities and an estimated left ventricular ejection fraction in the range of 10-25%. The acute anterior infarct pattern on ECG was seen in 4 patients however serial evaluation of creatine kinase and troponin I was abnormal in one patient with constrictive pericarditis. Low output syndrome and patient’s condition improved over the course 6-12 hours in 9 patients with medical management and IABP insertion and they were successfully weaned from inotropic support. A repeat echocardiogram showed improved left ventricular ejection fraction relative to preoperative value and without regional wall motion abnormalities in 7 patients. We postulate that in two patients this low output state may have been caused by a form of “myocarditis” induced by operative trauma during visceral pericardiectomy or myocardial injury by infection. In fact, the presence of myocarditis is often inferred from ST segment and T wave abnormalities on the ECG and in one case the clinical presentation may even simulate an acute myocardial infarction. The postoperative ECG changes in this patient then reverted to the baseline appearance that coincided with complete normalization of ventricular function. The ECG abnormalities associated with myocarditis are also usually transient and acute myocarditis simulating an acute myocardial infarction has a good prognosis, as with this patient. The radiation-induced cardiomyopathy caused POLCOS in two patients with lymphoma and angiosarcoma.

**Table 1:** Patients characteristics in study groups^a^

**Variable**	**Group A=409** **(Without polcos)**	**Group B=14** **(With polcos)**	**P-value**
Age (year, mean±SD)	45±12	48±9	0.887
Female sex	55%	71.4%	<0.001
BMI (mean±SD)	26±6	24±5	0.319
Constrictive pericarditis	5%	71.4%	0.949
Non constrictive pericarditis	95%	28.6 %	0.015
Inotropic drugs using	10%	100%	<0.001
IABP using (%)	0.2%	42.3 %	<0.001
Preoperative Ejection fraction (%)	50±12	35±7	<0.001
Calcification of pericardium (%)	4%	64.2%	0.012
POLCOS (%)	6.1%	35.7%	<0.001
Malignancy	2.2%	14.2%	0.0047
Radiotherapy	1.2%	14.2%	0.021
Supportive pericarditis	2.4%	7.1%	0.060

Abbreviations: LIMA, left internal mammary artery; POLCOS, Post operative low cardiac output. ^a^ Data are presented as mean± SD or percentage and number.

**Table 2:** Underlying cause of pericardial effusion in 423 patients

**Cause**	**Number of patients**
1-Malgnancy	105 (24.8%)
2- Renal failure	65 (15.3%)
3-Probably viral	10 (2.36%)
4- Connective tissue disease	50 (11.8%)
5- Post MI effusion	9 (1.8%)
6- Purulent effusion	12 (2.8%)
7- Dissection	2 (0.4%)
8- Tuberculosis	15 (3.5%)
9- Idiopathic	125 (29.5%)
10- Cardiac perforation from invasive procedure	6 (1.4%)
11- Hypothroidisem	10 (2.36%)

**Table 3:** Factors predicting polcos in hospital death in total patients by logistic regression analysis

Variables	Univariate			Multivariable		
	OR	95% CI	P	OR	95% CI	P
Age (year)	2.83	0.91 - 8.80	0.072	-	-	-
Purulent pericarditis	1.29	0.16 - 10.20	0.812	-	-	-
Malignancy	1.16	0.25 - 5.39	0.041	1.12	0.071- 5.25	0.000
Radiotherapy	0.41	0.05 - 3.22	0.397	2.01	0.87- 8.46	0.002
Constrictive pericarditis	4.67	1.55 - 14.13	0.006	3.55	0.97 - 13.02	0.055
Inotropic drugs using	0.44	0.06 - 3.47	0.438	0.96	0.93 - 0.99	0.001
IABP using (%)	0.95	0.92 - 0.98	0.002	1.23	0.07 - 0.735	0.043
Pre operative Ejection fraction (%)	0.15	-	0.404	2.06	0.123 - 0.456	0.045
Calcification of pericardium (%)	1.9	0.63 - 6.00	0.245	1.78	0.12 - 0.68	0.034

Abbreviations: BMI, body mass index; IABP, intra aortic balloon pump.

## Discussion

Pericardial effusion that cause tamponde, is a serious condition caused by the accumulation of fluid in the pericardial sac. Surgical placement of a subxiphoid tube is the preferred technique for draining of effusion in patients with acute sign and symptom that acutely developing pericardial tamponade, such as those with acute dissection or PCI complication. For patients with chronic and massive effusion and slowly developing pericardial tamponade, there are 2 principal methods: percutaneous catheter drainage and surgical tube drainage. In others center, the choice of drainage method depends on the cause of the effusion, the patient’s general health, the physician’s experience and preference, and the facilities available but in our center, cardiologist avoids percutaneous pericardiocetesis and almost all patients underwent surgical drainage.^19^ The causes of pericardial effusion change over time. At the time that we undertook this review, no causal evaluation of POLCOS after pericardial tamponade reliving had been performed recently in Iran. Moreover, our ability to determine specific causes in patients who had no apparent underlying disease was unknown, without access to pericardial tissue biopsy specimens. Pericardial effusion and constrictive pericarditis can occur after any pericardial disease process in developing countries. Idiopathic causes and cardiac surgery are two most predominant underlying etiologies followed by pericarditis and mediastinal radiation therapy. In developing and underdevelopment nations as well as in immunosuppressed patients, tuberculosis is major cause of constrictive pericarditis. Cause of PE includes: Specific causes of pericarditis include the following and are briefly reviewed below: 1- Idiopathic causes, 2- Infectious conditions, such as viral, bacterial, and tuberculosis infections, 3- Inflammatory disorders, such as RA, SLE, scleroderma, and rheumatic fever, 4- Metabolic disorders, such as renal failure, hypothyroidism, and hypercholesterolemia, 5- Cardiovascular disorders, such as acute MI, Dressler syndrome, and aortic dissection.^19-22^ Miscellaneous causes, such as iatrogenic, neoplasm, drugs, irradiation, cardiovascular procedures, and trauma. In this relatively small series, 14 patients (3.3%) had lowcardiac output syndrome within the first 24 postoperative hours,notwithstanding the “marked, sudden improvement in their hemodynamicstatus after drainage of the pericardial effusion. Despitemaximal inotropic support and IABP insertion, five of these fourteen patients (35.7%) diedseveral hours after the onset of the syndrome. The first patientwas a 27-year-old man with Hodgkin’s lymphoma of the mediastinal and retroperitoneallymph nodes who was subjected to chemotherapy and radiotherapy 6months before the operation. Her malignant disease was apparentlyunder control at the time of the operation, and his effusionwas not found to be malignant. The second patient was a 45-year-oldwoman with right atrial angiosarcoma and a malignant pericardial effusion, and low ejection fraction who was seen 1year after an open heart surgery and combined chemo-radiotherapy for right atrial sarcoma. The third patient was a 31-year-old man with purulent pericarditiscomplicated by cardiomyopathy and pericardial effusion resultingin cardiac tamponade. The fourth patient was a 40 year-old man tuberculosis, low EF and constrictive pericarditis, her tuberculosis was under control at time of pericardiectomy. Las, patient, was a 45-year-oldwoman with lupus erythematous, chronic pericardial effusion, pericardial calcification thatwas treated by prednisolone.In five of these fourteen patients there was a risk factor (low EF) that probablycontributed to their poor postoperative cardiac performance.The first and second patients probably had radiation-induced myocardialdamage, and the third, and fourth patients an infection-related cardiomyopathy. The otherpatient, however, had an apparently normal myocardium,as shown by preoperative echocardiography and operative findings however pericardiectomy of calcified pericardium causes myocardial injury as documented by postoperative cardiac enzymes rising.

In conclusion, in contrast to the experience of Moores et al^11^ and associates, we corroborate Neelakandan, Sunday, and Kanthimanthi’s^24-29^ clinical observation that low cardiac output syndrome cancomplicate any method of surgical pericardial drainage even in patientswith apparently normal myocardium and preoperative hemodynamicstatus. Therefore these patients require close postoperativemonitoring for the first 24 hours, preferably in the intensivecare unit, and aggressive treatment in case low cardiac outputdevelops. So far, we have not been able to define the causeof this highly lethal syndrome in patients with apparently normalmyocardium. However others patients have risk factors such as, cardiomyophaty, infection, calcification of pericardium, radiotherapy and malignancy induced low ejection fraction. The pathophysiologic mechanism might be the sameas that producing the syndrome in patients with constrictivepericarditis subjected to pericardiectomy.

## Conclusion

In conclusion we recommended that what may be the pathophysiologic mechanism of this lethal syndrome, it is logical that cardiologist or cardiac surgeon primarily consider gradual decompression of effusion by pericardiocentesis and after adaptation of myocardium by removal of effusion and improvement of hemodynamic condition, complete decompression of pericardium may be feasible. Treatment for this syndrome should be supportive with intraaortic balloon pump, inotropic support, which will result in recovery in some patients.

## Acknowledgments

The authors would like to acknowledge the kind efforts made by the statistical department and nurses at Imam Ali Hospital, Kermanshah University of Medical Sciences, Kermanshah, Iran.

## Ethical issues

Informed consent was obtained at admission, and the study protocol conformed to the ethical guidelines of the 1975 Declaration of Helsinki.

## Competing interests

Authors declare no conflict of interests in this study.
